# Gait control: a specific subdomain of executive function?

**DOI:** 10.1186/1743-0003-9-12

**Published:** 2012-02-09

**Authors:** Olivier Beauchet, Cédric Annweiler, Manuel Montero-Odasso, Bruno Fantino, François R Herrmann, Gilles Allali

**Affiliations:** 1 UPRES EA 2646, University of Angers, UNAM, France; 2 Department of Neuroscience, Division of Geriatric Medicine, Angers University Hospital, Angers, France; 3 Angers University Memory Clinic, Angers, France; 4 Department of Medicine, Division of Geriatric Medicine, the University of Western Ontario, London, Ontario, Canada; 5 Department of Internal Medicine, Rehabilitation and Geriatrics, Geneva University Hospitals and University of Geneva, Geneva, Switzerland; 6 Department of Neurology, Geneva University Hospital and University of Geneva, Geneva, Switzerland

**Keywords:** Gait disorders, Cognition, Motor impairment, Normal aging, Executive functions, Aging research

## Abstract

**Background:**

Few studies looked at the association between gait variability and executive subdomains (ESD). The aim of this study was to examine the association between ESD (i.e., information updating and monitoring) and stride time variability among healthy older adults.

**Methods:**

Seventy-eight healthy older adults (mean age 69.9 ± 0.9 years, 59% women) were divided into 3 groups according to stride time variability (STV) tertiles while steady state walking. Coefficient of variation of stride time was used as a marker of STV. Scores on cognitive tests evaluating information updating and monitoring (Digit Span test), mental shifting (Trail Making Test part A and part B) and cognitive inhibition (Stroop Color Word test) were used as measures of ESD.

**Results:**

The full adjusted and the stepwise backward logistic regression models showed that the highest tertile (i.e., the worst performance) of STV was only associated with lower Digit Span performance (Odds ratio = 0.78 with P = 0.020 and Odds ratio = 0.81 with P = 0.019).

**Conclusions:**

Information updating and monitoring are associated with STV in the sample of studied participants, suggesting that walking may be a complex motor task depending specifically of this subdomain of executive functions.

## Background

Walking is considered as an automatic motor activity mainly controled by subcortical and spinal regions [1]. Stride-to-stride variability of stride time (STV) is a measure of the reliability of lower limb movements and is a dependable marker of the rhythmic stepping mechanism [2,3]. A low STV (i.e.; under 5%) reflects an efficient gait control [3]. The past decade highlighted that cognitive functions, and in particular executive functions (EF), are associated with STV and play an important role in gait control even in routine walking condition [4-7]. EF are heterogeneous cognitive functions [8]. Based on Miyake's model, executive functions-mental set may be divided in 3 main separate executive subdomains (ESD), which are information updating and monitoring, mental shifting and cognitive inhibition [8]. Little is known about the association between these ESD and STV among healthy participants [7]. Safe gait requires to analyse an important flow of visual, vestibular and proprioceptive information, and to update these information in a short period of time, in order to adapt gait to the environmental conditions [3,5]. Thus, we hypothesized that STV could be associated with a specific ESD which is information updating and monitoring. The aim of this study was to examine whether there was an association between information updating and monitoring and STV among healthy older adults.

## Methods

### Participants

Between November 2009 and February 2011, 78 healthy older adults (mean age 69.9 ± 0.9 years, 59% women) were recruited from the GAIT (Gait and Alzheimer Interaction Tracking) study which is a cross-sectional study designed to compare gait characteristics of AD patients with healthy participants. In the present data, we focused on the gait characteristics of healthy participants.

### Clinical assessment

The number of drugs taken daily, height (m) and weight (kg) were recorded. Body mass index (BMI) was calculated as weight/height^2 ^(kg/m^2^). The participants were interviewed with standardized questionnaires gathering information about history of falls over the past year. A fall was defined as an event resulting in a person coming to rest unintentionally on the ground or at other lower level, not as the result of a major intrinsic event or an overwhelming hazard. Education level was evaluated with the number of years at school. The highest level of education corresponded to the highest number of years at school. ESD were evaluated based on Miyake's model that distinguishes mental shifting from information updating and cognitive inhibition [8]. We used the direct (i.e.; forward) and indirect (i.e.; backward) Digit Spans test for the evaluation of information updating and monitoring [9], the Trail Making Test (TMT) part A and part B for the evaluation of the mental shifting constituent [10], and the Stroop Color Word test for the evaluation of cognitive inhibition [11]. Digit spans test examines the ability to recall a sequence of numbers forward and backward in corrected order immediately after its presentation. It is a common measure of working memory that keeps several pieces of information active while we try to do something with them during a short period of time under 3 minutes. This management of information is based on monitoring and updating processes. The TMT evaluated two specific aspects of EF which are the visual attention and the task switching. This test requires a subject to "connect-the-dots" of 25 consecutive targets on a sheet of paper or a computer screen. Two versions are available: part A in which the targets are all numbers (1,2,3, etc.), and part B in which the subject alternates between numbers and letters (1, A, 2, B, etc.). The goal is to perform the test as quickly as possible, and the time taken to complete it is used as the primary performance metric. The Stroop color test is considered to specifically measure selective attention and cognitive inhibition. It is composed of three boards: board 1 shows color names written in black ink; board 2 shows color rectangles; board 3 shows color names written in color ink. Board after board, the subject must either read, or name the colors as quickly as possible, from right to left going to the following line at each end of the line. The number of words which has been read or color names found within 45 seconds is measured. The test is performed in the following order, with the following instructions: color naming (board showing color rectangles), reading of color names (board showing color names written in black) and interference situation (board showing color names written in color). For each board, the experimenter counts the number of words that have been read or colors named within the 45 seconds. The test-retest reliability of all these cognitive tests was moderate to high [12,13]. The total number of direct and indirect digit recalls in correct order, a ratio score of TMT (i.e., TMTB/TMTA) and a ratio score of Stroop (i.e., (''No Interference" [color])/''Interference" [color - word]) were used as outcomes.

### Gait recordings

STV was measured using SMTEC^® ^system (SMTEC^®^, Sport & Medical Technologies SA, Nyon Switzerland) [14]. The participants were asked to walk at their usual self-selected walking speed on a 10-meter walkway in a 20-meter corridor. According to the guidelines for spatio-temporal gait analysis in older adults and to assure that gait parameters were collected while steady state walking, participants started walking at least 2 meters before reaching the 10-meter walkway and completed their walk at least two meters beyond it [15]. Participants walked one trial in a quiet, well-lit corridor wearing their own footwear. Coefficient of variation (CoV) (CoV = (standard deviation/mean) × 100) of stride time was used as a marker of STV. Little is known about the test-retest reliability of STV but it has been recently reported that the immediate reliability of the CoV of stride time while single and dual tasking is poor [16].

### Statistics

The participants' baseline characteristics were summarized using means and standard deviations or frequencies and percentages, as appropriate. For the current analysis, participants were separated into 3 groups based on the tertilization of STV: lowest tertile (mean STV of 1.2 ± 0.3%; range [0.6-1.5]; n = 26), intermediate tertile (mean value of 1.8 ± 0.1%; range [1.6-2.0]; n = 26) and highest tertile (mean value of 2.9 ± 1.1%; range [2.1-6.4]; n = 26). Comparisons among groups were performed using one-way analysis of variance (ANOVA) with Bonferroni corrections or Chi-square test, as appropriate. Multiple logistic regression analyses were performed to specify the association between the highest STV tertile (dependent variable) and the ESD which was different across the 3 groups of participants (independent variable) adjusted for participants' baseline characteristics (age, gender, BMI, number of drugs taken per day, falls during the past year and education level). P-values less than 0.05 were considered as statistically significant. All statistics were performed using SPSS (version 17.0; SPSS, Inc., Chicago, IL).

### Standard Protocol Approvals, Registrations, and Patient Consents

Participants in the study were included after having given their written informed consent for research. The study was conducted in accordance with the ethical standards set forth in the Helsinki Declaration (1983). The entire study protocol was approved by the local Ethical Committee of Angers (France).

## Results

Table 1 showed the clinical characteristics of the studied sample of participants according to the tertiles of CoV of STV while walking alone in the studied population. Based on the established cut-off points from STV tertilization, gender (P = 0.003) and history of falls (P = 0.014) differed between groups, participants in the lowest tertile being less frequently female than those in the intermediate tertile (P = 0.001), and less frequently fallers than those in the intermediate (P = 0.004) and highest tertiles (P = 0.017). According to ESD assessment, only performance on Digit Span test differed between groups (P = 0.004), those in the lowest tertile of STV (i.e., the best gait performance) having enumerated more figures than those in the highest tertile (P = 0.004). There was no significant difference for the others characteristics.

**Table 1:** Clinical characteristics of the studied sample of participants according to the tertiles of stride-to-stride variability of stride time (STV) while walking alone (n = 78)

	Tertiles of STV			P-Value*			
	
	Lowest (n = 26)	Intermediate (n = 26)	Highest (n = 26)	Overall	Lowest versus intermediate	Lowest versus highest	Intermediate versus highest
Age (years), mean ± SD	70.0 ± 1.0	69.7 ± 0.7	69.8 ± 0.8	0.510	-	**-**	**-**
Female, n (%)	9 (34.6)	21 (80.8)	16 (61.5)	**0.003**	**0.001**	0.052	0.126
BMI (kg/m^2^), mean ± SD	24.8 ± 2.6	25.6 ± 3.3	26.6 ± 4.4	0.187	-	-	**-**
Number of drugs taken daily, mean ± SD	2.0 ± 1.5	2.5 ± 2.0	2.7 ± 2.5	0.406	**-**	-	-
Falls during the past year, n (%)	2 (7.7)	11 (42.3)	9 (34.6)	**0.014**	**0.004**	**0.017**	0.569
Education level† (years), mean ± SD	11.8 ± 3.3	10.9 ± 3.2	11.8 ± 3.7	0.550	**-**	**-**	**-**
Executive subdomain performance							
Digit Span score‡, mean ± SD	15.5 ± 3.6	13.5 ± 3.2	12.7 ± 2.3	**0.004**	0.060	**0.004**	1.000
Ratio score TMTB/TMTA#, mean ± SD	2.1 ± 0.6	2.2 ± 0.6	2.0 ± 0.5	0.486	-	**-**	-
Ratio score Stroop Part III/ Part I, mean ± SD	2.9 ± 0.8	3.2 ± 0.8	3.2 ± 1.6	0.530	**-**	**-**	**-**

BMI: Body mass index

TMTA: Trail Making Test A

TMTB: Trail Making Test B

* Comparison based on oneway ANOVA, Kruskal-Wallis test with Bonferroni corrections or Chi-square test, as appropriate

†: Assessed with the number of years at school

‡: Total number of digits that a subject can absorb and recall in correct forward and backward serial orders after hearing them

#: Times to "connect-the-dots" as quickly as possible of 25 consecutive targets on a sheet of paper; part A the targets are numbers, and part B alternated numbers and letters

: Ratio score Stroop Color Word test Part III/ Part I (Part I corresponding to time to name color, and part III corresponding to time to name the color of incongruent color words)

P significant (< 0.05) indicated in bold.

As shown in Table 2 the full adjusted and the stepwise backward logistic regression models showed that the highest STV tertile was associated with lower performance on Digit Span (Odds ratio [OR] = 0.78 with P = 0.020 and OR = 0.81 with P = 0.019, respectively). There was no association between STV and the other covariates.

**Table 2:** Multiple logistic regression models showing the association between the highest tertile of stride-to-stride variability of stride time (dependent variable) and Digit Span score (independent variable) adjusted for clinical characteristics (n = 78)

	Full adjusted model			Stepwise backward model		
	
	OR	95% CI	P-value	OR	95% CI	P-value
Digit Span score *	**0.78**	**[0.63;0.96]**	**0.020**	**0.81**	**[0.68;0.97]**	**0.019**
Age	1.02	[0.50;2.10]	0.954	**-**	**-**	**-**
Female	1.05	[0.32;3.4]	0.932	**-**	**-**	**-**
BMI	1.09	[0.94;1.27]	0.253	**-**	**-**	**-**
Education level †	1.11	[0.95;1.30]	0.182	**-**	**-**	**-**
Number of drugs taken daily	1.00	[0.77;1.30]	0.984	**-**	**-**	**-**
History of falls ‡	2.07	[0.57;7.54]	0.270	-	-	-

OR: Odds ratio

CI: confident interval

BMI: body mass index

*: Total number of digits that a subject can absorb and recall in correct forward and backward serial orders after hearing them

†: Assessed with the number of years at school

‡: Falls in the previous year

Odds ratio significant (i.e.; < 0.05) indicated in bold.

## Discussion

Our results show that gait control assessed by the STV was associated with a specific ESD, the information updating and monitoring assessed with the Digit Span test. More precisely, an increase of one unit in the variability of stride time was significantly associated with a 1.28 (1/0.78) fold decrease one unit in digit span score, which corresponds to a 28% decrease.

Previous studies have already shown an independent association between EF performance and gait [4-7,17]. Nevertheless, most of them focused on participants with neurodegenerative diseases such as patients with the behavioural variant of the frontotemporal degeneration [6]. Here, we showed that gait of healthy older adults is a motor task requiring the involvement of higher-level cognitive input even in steady-state while usual walking. Few studies have explored the association of EF with gait performance among healthy older adults [4,5,17]. Some of them found that a low performance on global EF was associated with a low gait speed and its rate of gait speed decline, but none found that the impairment in a specific ESD could account for slowing of usual gait [4,17]. Difference with our findings could be due to the use of a different gait characteristic. STV compared to gait speed is indeed a specific temporal gait parameter which has been related to gait control [3]. Furthermore, in a previous study comparing the relationship between gait control and higher-level cognitive resources, Hausdorff et al. also showed that STV was specifically associated with EF, but not with memory or global cognitive functions [5]. The involvement of EF we observed among healthy older adults could be explained by age-related changes in sensorimotor system leading to a decrement in automaticity compensated by an increased involvement of EF to properly process all sensorimotor information. Recently, this hypothesis was reinforced by a MRI study which showed that white matter hyperintensity load correlates with EF dysfunctions as well as gait and posture measures [18]. The association between STV and updating and monitoring function shown in our study is not surprising because walking requires processing a flow of information in a short period to adapt gait to the environmental conditions. These results open new perspectives in the improvement of fall prediction and of gait rehabilitation. Indeed, because dementia-related gait disorders have been associated to central misprocessing of information due to EF dysfunctions, it could be suggested that impairment of updating and monitoring shown in demented older adults may be used as a predictor of falls. Furthermore, gait rehabilitation based on an improvement of updating and monitoring function could be a new way to prevent falls.

The main limitation of our study was the relatively small number of participants included in the current analysis. In addition, although we were able to control for many characteristics, residual potential confounders may still remain.

## Conclusions

Taken together, previous and our findings provide a new insight into gait control. Even relatively simple, steady-state walking should be considered more as a complex motor task than an automated and rhythmic motor task. Based on Miyake's model, our results highlighted that gait control is associated with one specific ESD, the information updating and monitoring. Future investigations should investigate whether this association is also observed among younger participants or is a consequence of aging.

## Competing interests

The authors declare that they have no competing interests. There were not any financial and personal relationships with other people or organization that could influence this study.

## Authors' contributions

OB has full access to the data in the study and takes responsibility for the integrity of the data and the accuracy of the data analyses. Study concept and design: GA and OB. Acquisition of data: CA and BF. Analysis and interpretation of data: OB, GA, FRH, VD, RWK and CA. Drafting of the manuscript: OB, CA and GA. Critical revision of the manuscript for important intellectual content: MMO, FRH and BF. Statistical expertise: FRH. Administrative, technical, or material support: CA. Study supervision: OB and GA. All authors read and approved the final manuscript.
